# Overview of Cadmium Thyroid Disrupting Effects and Mechanisms

**DOI:** 10.3390/ijms19051501

**Published:** 2018-05-17

**Authors:** Aleksandra Buha, Vesna Matovic, Biljana Antonijevic, Zorica Bulat, Marijana Curcic, Elisavet A. Renieri, Aristidis M. Tsatsakis, Amie Schweitzer, David Wallace

**Affiliations:** 1 Department of Toxicology “Akademik Danilo Soldatović”, University of Belgrade-Faculty of Pharmacy, 11000 Belgrade, Serbia; vesna.matovic@pharmacy.bg.ac.rs (V.M.); abiljana@pharmacy.bg.ac.rs (B.A.); zorica.bulat@pharmacy.bg.ac.rs (Z.B.); makitox@pharmacy.bg.ac.rs (M.C.); 2 Laboratory of Toxicology, University of Crete, Medical School, 71003 Crete, Greece; medp2011622@med.uoc.gr (E.A.R.); tsatsaka@uoc.gr (A.M.T.); 3 School of Biomedical Science, Oklahoma State University Center for Health Sciences, Tulsa, OK 74107, USA; ajoncko@ostatemail.okstate.edu (A.S.); david.wallace@okstate.edu (D.W.)

**Keywords:** cadmium, endocrine disruption, thyroid gland, mechanisms

## Abstract

Humans are exposed to a significant number of chemicals that are suspected to produce disturbances in hormone homeostasis. Hence, in recent decades, there has been a growing interest in endocrine disruptive chemicals. One of the alleged thyroid disrupting substances is cadmium (Cd), a ubiquitous toxic metal shown to act as a thyroid disruptor and carcinogen in both animals and humans. Multiple PubMed searches with core keywords were performed to identify and evaluate appropriate studies which revealed literature suggesting evidence for the link between exposure to Cd and histological and metabolic changes in the thyroid gland. Furthermore, Cd influence on thyroid homeostasis at the peripheral level has also been hypothesized. Both in vivo and in vitro studies revealed that a Cd exposure at environmentally relevant concentrations results in biphasic Cd dose-thyroid response relationships. Development of thyroid tumors following exposure to Cd has been studied mainly using in vitro methodologies. In the thyroid, Cd has been shown to activate or stimulate the activity of various factors, leading to increased cell proliferation and a reduction in normal apoptotic activity. Evidence establishing the association between Cd and thyroid disruption remains ambiguous, with further studies needed to elucidate the issue and improve our understanding of Cd-mediated effects on the thyroid gland.

## 1. Introduction

Defined as an exogenous chemical or a mixture of chemicals that interfere with any aspect of endogenous hormone action, endocrine disruptors (ED) have risen to scientific and public attention within the past few decades [1]. Obesity, morbidity, and carcinogenicity induced by ED exposure are an evolving reality [2]. While characterization of ED with estrogenic and androgenic activities is well established [3], very little is known about environmental stressors that exert effects on the thyroid system.

The thyroid system plays a pivotal role in the body homeostasis and functioning of the nervous, cardiovascular and reproductive systems, and of body growth control [4,5,6]; hence, putative thyroid disruptors can cause significant impairments in living organisms. Thyroid function is controlled by the hypothalamic-pituitary-thyroid axis, with the mediation of Thyrotropin-Releasing Hormone (TRH), Thyroid-Stimulating Hormone (TSH), thyroxine (T4), and triiodothyronine (T3). These hormones are linked by complex negative feedback mechanisms. Namely, reactions necessary to form T3 and T4 in follicular cells in the thyroid gland are influenced and controlled by the pituitary TSH while its secretion is controlled by the negative feedback of circulating levels of T4 and T3 as well as by the influence of hypothalamic THR [7]. The effects of thyroid hormones mainly depend on intracellular concentrations of T3, which is the biologically active form and its binding to nuclear receptor (TR). Most circulated T3 is created through extrathyroidal deiodination of T4 that mainly occurs in the liver [8]. Thyroid disorders are the common glandular disorders of the endocrine system [9] and include hypothyroidism, hyperthyroidism, goiter and other iodine deficiency disorders, such as Hashimoto and thyroid cancer. In recent decades, the prevalence of autoimmune thyroid diseases and thyroid cancers has increased. The elevation of specific antibodies, used as standard laboratory biomarkers for the autoimmune thyroid disease diagnosis, is of great concern nowadays [10]. A recent study on Danish twins demonstrated that apart from genetic factors, another critical factor responsible for the presence of these biomarkers are environmental factors, which account for 20–30% [11]. Furthermore, thyroid cancer has become the fifth most common cancer amongst women [12], and recent studies have pointed to the important role of environmental factors, especially heavy metals, in promoting thyroid cancer incidence [13].

Cadmium (Cd) is one metal with established endocrine disrupting activities. This toxic metal is widespread in the environment both naturally and as a pollutant emanating from industrial, agricultural and other sources [14]. Significant sources of Cd exposure for the general population include diet and tobacco smoking. Drinking water as a source of Cd is limited both in industrialized and non-industrialized areas [15]. Consequently, people are ubiquitously exposed to low doses of Cd [14]. The main routes of exposure are via the digestive tract and inhalation. The most important Cd sites of accumulation are in the liver, kidneys, and muscles. Another possible site of Cd deposition in the body is the thyroid gland due to the presence of cysteine-rich proteins, metallothioneins (MT), which bind Cd and represent a potent intracellular Cd detoxifier [16]. It has been observed that Cd concentrations in thyroid gland are three times higher in people living in areas polluted with Cd when compared to people residing in nonpolluted areas [17]. Because of Cd binding to molecules rich in sulfhydryl groups such as MT, glutathione, etc. and slow excretion, the Cd biological half-life is between 5 and 30 years. Thus, exposure to Cd even at environmentally low levels over time is associated with a plethora of toxic effects on kidneys, the liver, bones, testes and the cardiovascular system [18,19,20]. Novel investigations point to its endocrine disrupting properties, suggesting the role of Cd in diabetes mellitus [21], and its estrogenic activity [22,23]. Moreover, Cd has been classified as a group 1 human carcinogen with sufficient evidence for the lung and limited evidence for the prostate and kidneys [24]. Recent studies also provide evidence of Cd association with other types of cancers such as breast [25], pancreatic [26], urinary bladder cancer [27], etc.

This review will re-assess and provide an update on the association between Cd exposure and disruption of thyroid function homeostasis as well as on the possible role of Cd in thyroid cancer development.

## 2. Cadmium Effects on Thyroid Function

Endocrine toxicity can result in hyperfunction or hypofunction of the gland. Chemically induced thyrotoxicity is reflected by imbalanced plasma T4 and/or T3 and/or TSH levels, which are commonly used as reliable indicators of the thyroid function in humans and experimental animals. Changes in serum hormone levels can reflect disturbances in their glandular synthesis and/or secretion as well as disorders in their extra-thyroidal peripheral metabolism. Another marker of thyrotoxicity is structural damage to thyroid tissues, i.e., thyroid hypertrophy or hyperplasia. Evidence for Cd thyrotoxicity has been reported in multiple previous studies.

### 2.1. Human Studies

Much of the work that has been done to date include retrospective human studies which have correlated Cd exposure to alterations in thyroid hormone function [28,29,30,31]. In humans, prior studies have found a positive correlation between urinary Cd and all thyroid hormones as well as thyroglobulin (Tg) [28,29]. Chen et al. [28] analyzed the results of the National Health and Nutrition Examination Survey (NHANES) 2007–2008 data collected in the general US population with higher environmental exposure levels. Higher thyroid hormone levels and Tg levels in adults were associated with increased Cd urinary levels, i.e., higher Cd exposure. Levels of TSH, on the other hand, were not consistently associated with Cd exposure. These results need further clarification since they can be attributed to possible chance or bias. It is worth noting that observed associations were relatively weak on the individual level, with about 1–4% change in thyroid hormones per interquartile range increase in Cd. In another study using NHANES 2007–2008 data [29], but using different statistical methods of analysis and smaller sample size, urinary Cd levels were also correlated with both increased T3 and T4 levels. Furthermore, this study found elevated blood Cd levels associated with decreased TSH levels, suggesting this inverse relationship between Cd and TSH indicates overt thyroid disease due to Cd exposure. Blood Cd correlated to lower TSH levels and unchanged T3 and T4 levels, which is a pattern of sub-clinical primary hyperthyroidism, while urinary Cd was associated with higher T3 and T4 levels but unchanged TSH levels, suggestive of secondary hyperthyroidism. Comparing blood versus urine Cd levels, urine Cd levels represent total body burden while blood levels reflect recent exposures [14]. Based on the previous data presented, we can hypothesize that thyroid hormone levels are strongly associated with total Cd body burden, while changes in TSH levels are reflective of recent exposure to Cd. Recently, a study using the NHANES data was able to distinguish correlations between urinary Cd and thyroid hormone concentration [30]. Adding to the existing literature, the authors included interaction effects between various trace and toxic metals in the statistical model and used separate models for males and females. Positive statistically significant correlations were found between Cd and FT3 levels and Cd and Tg levels, but for males only, pointing to gender-based differences in the response of thyroid function to Cd exposure. Similarly, Luo and Hendryx [31] find that Cd exposure on thyroid axis may differ by sex in a study which analyzed participants from NHANES from 2007–2010. A positive correlation between Cd exposure and log-Tg was observed for both sexes, while a positive correlation between Cd exposure and total T3 levels was observed only among males. These gender-based differences are difficult to explain and may be the consequence of toxicokinetics and hormonal differences between sexes that certainly influence observed responses. Studies by Rosati et al. [32] and Jurdziak et al. [33] have added the factor of occupational exposure to Cd. An outdoor study on 277 individuals of both sexes exposed to urban pollutants has found a negative correlation between urinary Cd levels and free T3 and T4 levels and a positive correlation between Cd urinary levels and TSH levels, showing that occupational exposure to the low Cd concentration present in urban air affects thyroid function [32]. Another study on workers occupationally exposed to Pb, Cd, and arsenic also confirmed that higher blood Cd concentrations amplify the risk of abnormal hormonal thyroid function evaluated by elevated TSH levels [33]. The regression coefficient for blood Cd and TSH concentrations was 1.26, suggesting that elevated blood Cd correlates with increased TSH levels. However, in a recent study on the effects of lead (Pb) and Cd on the thyroid function of welders , no changes in T4, T3 and TSH in relation to increased Cd levels were found [34]. Similarly, a study on 219 male patients in an infertility clinic revealed no significant association between Cd blood concentrations and TSH levels [35]. Collectively, human studies have yielded conflicting data, leading to increased confusion about the relationships between Cd and thyroid hormones. The difficulty in interpreting these findings is due to variations in patient selection, as depending on the objective of the study, unclear consideration for pre-existing thyroid conditions. The means for measuring free and bound hormones differ between studies, possibly leading to increased variability in the quantification of free and bound T3 and T4. The times at which T3, T4, TSH and Tg are measured are also important variables. Shorter durations of Cd exposure would result in less robust changes in these indices, thus weakening any correlation between Cd and hormone levels. The quantification method for Cd, in the urine or plasma would have a profound effect on determining relationships between Cd and thyroid hormone levels. One possible explanation would be that a combination of slightly elevated hormone metabolism in the periphery coupled with an increased expression of Tg would result in a reduction of free T3 and free T4, whereas the bound concentrations of these hormones may be unchanged or increased. In response to reduced free T3 and T4, the pituitary is activated to increase the production and release of TSH to compensate for apparent reductions in thyroid hormone concentrations. Differences in exposure times and means of exposure is clearly one more important factor contributing to the conflicting results. Occupational versus environmental exposures, as well as the route of administration, also affect previous findings. Questions as to whether Cd was inhaled, ingested, or absorbed are important considerations for the long-term toxicokinetics of Cd. We can see a relationship between acute and chronic exposure and which thyroid index is affected, such as T3 or T4 levels compared to TSH levels. Only a highly controlled and regulated study would be able to account for these variables.

Novel studies in humans examined the possible role of Cd in autoimmune processes in the thyroid gland. The study based on the 2014 SPECT-China study (including 5628 Chinese adults) showed the relation of blood Cd levels and sera antibodies to thyroid proteins and thyroid dysfunction reflected by total T3, total T4 and TSH levels [10]. Blood Cd levels positively correlated to higher TSH and hypothyroid status in women and higher antibodies levels in women. These observations on sex-biased thyroid autoimmunity induction were explained by the ability of Cd to activate estrogen receptors proved both in vivo and in vitro [22], since estrogen has regulatory effects on innate immune cells [36]. Moreover, in a cross-sectional case-control study investigating the status of some essential elements and some toxic metals and metalloides (among which was Cd) in the blood of 22 females, patients with Hashimoto thyroiditis and overt hypothyroidism were found to possess higher levels of Cd compared to Cd levels in 55 healthy females [37]. The ability of cadmium to interfere with thyroid hormone levels is unclear in children. In a study involving three-year-old children from an informal e-waste recycling area in China, the effects of thyroid disruption on the mental development in children and the role of Cd or Pb blood levels in these processes were evaluated [38]. Significantly higher Cd and Pb blood levels as well as higher FT4 and TSH concentrations and reduced cognitive and language scores were observed in children residing near an e-waste area in comparison to the reference group. Nevertheless, Cd levels did not significantly correlate with higher levels of FT4 and TSH, nor did they observe a correlation with scores. On the other hand, another study reported the presence of a significant negative correlation between umbilical cord Cd concentration and neonatal TSH levels [39]. This study indicated the possible effects of in utero Cd exposure on thyroid function status of newborns.

Human studies have shown that thyroid function may be disrupted by either occupational or low-level environmental exposure within the general population. However, the observed outcomes are complex and often conflicting, probably mediated by sex differences, and by Cd exposure duration and levels. The outcomes could also be affected by the fact that observed populations are a mixture of those with a normal thyroid function, and those with potential undiagnosed or subclinical thyroid diseases. These results point to a non-linear dose-response relation (see Section 4). Another important point is the fact that even though exposure to Cd may not produce a significant clinical effect (a change occurring is not outside the normal laboratory range), small, but statistically significant changes can become clinically significant as well over the period of continual exposure. The difficulty in controlling for multiple factors that affect the toxicokinetics and toxicity of Cd is a major challenge in human studies. Increasing evidence suggests that gender, specifically the sex-steroid hormones estrogen/testosterone, exerts influence over Cd toxicity. In addition, the physiological characteristics of the subject, weight, fat composition, size of total body water, hepatic and renal function, and age of the subject all influence the toxicity of Cd. It is clear that there is a space for additional research that would shed light on the complex nature and mechanisms of Cd-thyroid disruption in humans.

### 2.2. Animal Studies

#### 2.2.1. Studies in Lower Vertebrates

Investigations of Cd thyrotoxicity on different fish species demonstrated altered fish thyroid cascade as a result of both acute and chronic Cd exposure, as reviewed by Nugegoda and Kibria [40]. In a study conducted by Hontela et al. [41] juvenile rainbow trout (*O. mykiss*) were exposed to 0, 0.4, 0.8 and 2.4 mg Cd/L for 4, 24, 96 h and to 0, 0.4 and 0.8 mg Cd/L for a week through their water. Levels of T4 in juvenile rainbow trout (O. mykiss) were increased following short-term exposure (for 2 and 4 h) at all doses, while subacute exposure led to the decrease in T4 levels [41]. Plasma T3 levels remained unchanged in exposed fish. A study performed in tilapia (*O. niloticus*) showed a significant and sustained reduction in T3 plasma concentrations following acute exposure to sublethal Cd concentrations (25 mg/L of CdCl_2_ for 24, 48 and 96 h) [42]. A significant and sustained decrease in plasma T3 concentrations was observed at all investigated time points, while no changes were observed in T4 levels. Reductions in T3 plasma concentration were not attributed to thyroid-mediated changes, but to other peripheral effects such as increased T3 metabolism. However, a study in walking catfish (*C. batrachus*) revealed the reduction in epithelial height of the thyroid follicles as a consequence of Cd subacute exposure [43], indicating Cd direct effects on thyroid gland. Exposure to Cd (0.5 and 2.5 mg/L) for four (4) days in the rare Chinese minnow (*G. rarus*) larvae caused significantly decreased T4 levels in the group treated with a higher Cd level. Both thyroglobulin and TSH mRNA was upregulated, but gene expression for thyroid hormone receptors α and β were both downregulated. Altered gene expression related to hypothalamic-pituitary-thyroid (HPT) axis for both exposure levels detected in this study indicated that the Cd endocrine toxicity might be a result of HPT axis disruption in fish [44]. Longer, subacute exposure (i.e., 30 days) to CdCl_2_ in water (10 and 25 µg Cd/L for adults, and 1 and 5 µg/L for juveniles) decreased both T4 and T3 levels in juvenile and adult rainbow trout, but this decrease was not statistically significant [45]. It can be concluded that the type of exposure, the dose and the duration of exposure, as well as the fish species and used protocols determine whether Cd will produce an increase, decrease or no effect on thyroid hormones plasma levels. However, some form of the thyroid cascade alteration will occur as a result of Cd exposure. Summary of reviewed studies is given in Table 1.

#### 2.2.2. Studies in Higher Vertebrates (Mammals)

Studies conducted on rats showed the effects of Cd on the gland weight, histopathology, and thyroid hormone status. Various directions of changes in the gland and hormone levels as well as the lack of changes were reported depending on Cd level and exposure duration.

In a study conducted by Hammouda et al. [46], oral treatment with 200 ppm Cd (as CdCl_2_) in their drinking water for 35 days resulted in significantly decreased T4 serum levels in Wistar albino rats. Serum TSH levels were significantly increased in Cd-exposed rats, possibly because of feedback control mechanisms. Based on this result, the authors suggested that Cd did not affect directly pituitary TSH synthesis and/or secretion. Similarly, adult male Sprague-Dawley rats treated with 50 mg Cd/L as CdCl_2_ added to the drinking water daily for 4 weeks had increased TSH levels compared with the control group [47]. This elevation in TSH levels was explained as a response to decreased T3 and T4 levels also observed in the study. However, another study proposed a direct effect of Cd on the pituitary gland. Namely, plasma levels of TSH in adult male Sprague-Dawley rats exposed to CdCl_2_ in the drinking water at different Cd doses (5, 10, 25, 50, and 100 ppm) for one month were increased, but the dose-dependency was not observed. However, since Cd concentration was increased in the pituitary gland, a direct effect was speculated [48]. Cadmium effects on the daily secretory pattern of TSH was also investigated in adult male Sprague-Dawley rats treated with two different Cd doses (25 and 50 mg/L CdCl_2_) in their drinking water for 30 days [49]. Although the daily pattern of TSH was unchanged, the median TSH levels around the clock were increased most likely because of pituitary compensatory mechanisms. Another study has reported that TSH levels are reduced following Cd exposure [50]. In this study, rats were given CdCl_2_ daily doses of 0.55 and 2.19 mg/L in drinking water for three months. Decreased T3, T4 and TSH levels were observed in both exposed groups, suggesting the ability of Cd to prevent TSH elevation in conditions of decreased T3 and T4, i.e., Cd interference with pituitary regulation of thyroid hormone production and release. Similarly, serum concentration levels of TSH were not statistically changed after chronic Cd exposure (12 months in drinking water at the concentration of 5 and 50 mg/L) in female rats [51]. The exposure to either Cd concentration did not alter serum T3 concentration, while the higher concentration provoked a decrease in T4 levels. The lack of a significant TSH response to the decreased serum T4 level was explained by Cd interference with the pituitary regulation of thyroid hormones production and secretion. Collectively, these studies, although performed in different rat strains, using different doses and duration of exposure, showed the ability of Cd to change TSH levels when acting on the thyroid and/or pituitary level. Concerning T3 and T4 levels, most rodent studies have reported an apparent generalized decrease in serum T3 and T4 levels. Buha et al. [52] investigated the effects of 6 different doses of Cd orally administrated to Wistar rats and showed more a profound decrease in T3 serum levels compared to decreases in serum T4 levels. Furthermore, the authors demonstrated a dose-response relationship for the Cd-mediated effects on both hormones and based on low calculated levels of benchmark dose for T3 proposed precisely this parameter as the point of departure for deriving different health-based guidance values. However, the lack of Cd interference with thyroid hormone levels observed in some of the aforementioned studies may be a reflection of the different experiment methodologies used for the measurement of T3, T4 and TSH in rodents. In addition to use of different rat strains, doses and duration of exposure to Cd, the type of administration in these studies was also different, i.e., some studies administered Cd via oral gavage, and others with salts mixed within the feed, while different studies included the salt in the animal’s drinking water. Hence, observed discrepancies could reflect different responses to these administration types. Indeed, although all these treatments being orally-based, they differ in absorption rate, distribution and availability of Cd. A summary of studies analyzing Cd effects on the thyroid function in rats is given in Table 1.

Another important issue relating to thyroid-disrupting substances and ED substances is the fact that humans are more often exposed to multiple disrupting substances simultaneously and these substances can exert additive or even synergistic effects [53]. Particular attention was given to mixtures consisting of metals and persistent organic pollutants, especially those which are highly toxic compounds and already established disruptors of thyroid function such as polychlorinated biphenyls (PCB) [54,55,56] and polybrominated diphenyl ethers [57,58,59]. Study on mixtures of Cd and PCB [52] revealed possible synergistic effects of Cd and PCBs on T3 and T4 levels after 28 days of oral administration of a Cd-PCB mixture in Wistar rats. Subacute oral treatment with a mixture of Cd and decabrominated diphenyl ether was more potent at disturbing thyroid hormone homeostasis (measured by T3 and T4 serum levels) in rats than the same doses of individual chemicals [60]. A complex, environmentally relevant mixture of 16 common organochlorines, several chlorinated benzenes, and metal contaminants produced some disturbances on the HPT axis in sexually mature male rats after 70 daily treatments by gavage [61]. Exposure to mixtures of organochlorines and Cd resulted in complex changes, but alterations in TSH levels and hepatic deiodinase activity being the most sensitive and reproducible. These studies raise an important issue of the combined effects of low doses of ubiquitous thyroid-disturbing substances that cannot be easily predicted.

## 3. Mechanisms of Cadmium Thyrotoxicity

Endocrine toxicity comprises of two types of toxicities that are often associated with each other. While primary endocrine toxicity involves the direct effect of a chemical on the target gland, secondary endocrine toxicity represents effects detected in an endocrine gland because of toxic effects in the endocrine axis.

### 3.1. Primary Endocrine Toxicity

In vivo studies on rats revealed disturbances in thyroid gland caused by Cd administration. The direct toxic effects of Cd on thyroid gland were studied in the study by Yoshizuka et al. [62]. Intraperitoneal administration of Cd-sulphate for only 4 days caused Cd accumulation in the swollen mitochondria of the thyroid follicular epithelial cells and deterioration of the rough-surfaced endoplasmatic reticulum; this can progress into the disturbances of the active absorption of colloid droplets. Moreover, the disappearance of Tg-secreting granules was noted in follicular epithelial cells, which in turn may cause a significant decrease of the thyroid hormones into circulation. Chronic Cd exposure (4 weeks) in thyroid follicular cells of female rats influenced both the structure and function of these cells (manifested by flattening follicular cells, enlargement of interstitial tissue between follicles) dose-dependently, regardless of the low Cd retention in the cell [51]. In a study conducted by Hammouda et al. [46], oral Cd treatment for 5 weeks resulted in its accumulation in the thyroid gland accompanied by an increase of the relative thyroid weight. After a 3 month long treatment with 15 mg/kg Cd dissolved as CdCl_2_ in drinking water, histopathological and immunohistochemical research of the thyroid gland of Wistar rats revealed a spectrum of preneoplastic changes on thyroid folliculi and verified Tg hyposecretion and an absence of secretion in areas of adenomatoid follicular hyperplasia [63]. Investigations in lower vertebrates also confirmed Cd direct effects on the thyroid gland. Namely, chronic Cd exposure with higher Cd concentrations (50, 100, and 500 µg/L) in Chinese amphibian toad (*B. gargarizans*) tadpoles caused histopathological changes of the thyroid gland reflected by follicular cell hyperplasia and malformation [64]. Amphibians are regarded as a good indicator for aquatic environmental changes because of their gill and skin permeability and vulnerability, and their metamorphosis is considered to be regulated by hormones produced by the thyroid gland. A series of validation studies and reports have revealed that the histopathology of the amphibian thyroid gland is very sensitive and reliable for detection of antithyroidal activities of various chemicals, as reviewed by Miyata and Ose [65]. In the same review however, the authors stressed that because amphibian thyroid glands change continuously throughout metamorphosis, there are some difficulties, which should be taken into account while performing histopathological examination. Hence, experienced pathologists who are familiar with normal thyroid histology, thyroid gland physiology and general responses of the thyroid gland to agonists or antagonists must perform these examinations.

The direct effect of Cd on the thyroid gland can be proposed in all studies that showed decreased T4 levels but unchanged T3 levels, since the thyroid gland is the only organ involved in T4 synthesis and its changes in serum levels suggest that Cd influences the production and/or secretion of this hormone by follicular cells. Possible mechanisms behind this Cd effect on the thyroid gland can be oxidative stress (i.e., oxidative phosphorylation disorders in mitochondria of follicular cells) as suggested by many authors [46,62,66].

**Figure 1:**
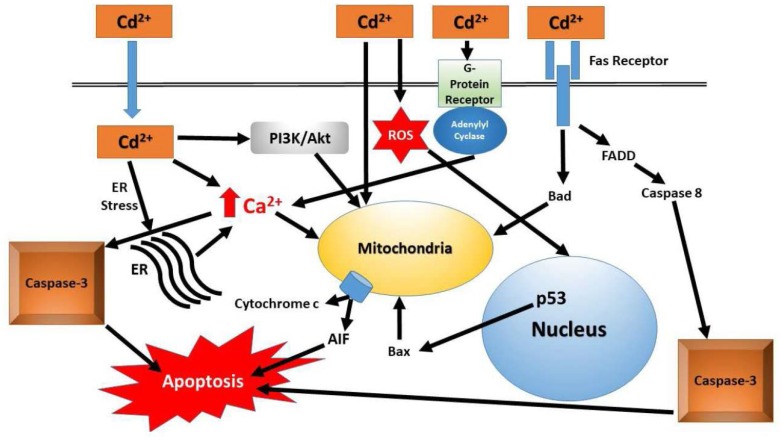
Schematic of Cd-mediated cellular effects. The primary pathways are to induce apoptosis when there is a cellular challenge from Cd exposure. When the ability of the cells to efficiently enter apoptosis for repair, or if apoptosis is blocked, the cell will continue to proliferate and perpetuate the genetic damage caused by oxidative stress. Damage to the mitochondrial will occur over time, membrane permeability will change and normal respiration resulting in the generation of ATP will cease. Abbreviations: ROS = Reactive Oxygen Species; FADD = Fas-Associated protein with Death Domain; ER = Endoplasmic Reticulum; AIF = Apoptosis-Inducing Factor; PI3K/Akt = Phosphoinositide 3-Kinase/Protein Kinase B.

Further investigations of mechanisms of direct Cd thyroid toxicity were conducted in vitro. Early studies in non-thyroid cells (liver, lymph, lung, blood, etc.) have established the foundation for future thyroid studies in vitro. Investigators have examined the effects of plasma Cd on the function of lymphocytes [67,68]. Changes in lymphocyte function involve changes in both mitochondrial function and apoptosis. The death receptor “Fas” is upregulated and there is an increase in CD95/Fas complex [68]. Fas can function through two pathways, apoptosis and mitochondrial, and therefore appears to be pivotal in cellular death. Lymphocyte exposure to Cd results in an increased expression of caspase-3 and p53, further supporting the hypothesis that Cd exerts cellular damage through changes in apoptotic pathways [68]. Upregulation of p53 leads to an upregulation in the pro-apoptotic gene, Bak [68]. The second pathway activated by Fas involves changes in mitochondrial function and generation of ROS by the mitochondria [67]. Additionally, acute exposure to Cd resulted in multiple alterations in lymphocyte function that includes ROS generation, fragmentation of DNA, and DNA mutations leading to cell death through apoptosis and necrosis. Cd clearly interacts with multiple protein systems involved in modulating oxidative stress responses [67]. Alkharashi et al. [67] presented an elegant set of studies in which they examined the gene expression, protein expression, and mitochondrial function of cells exposed to varying concentrations of Cd over 48 h. They report altered multiple expression patterns (gene and protein) in a Cd concentration-dependent fashion. The authors presented a Cd-associated protein-interaction network image that clearly indicates a complex interaction network between Cd and many proteins. Induction of oxidative stress-related proteins was also produced in lung fibroblasts and oligodendrocytes, with a profile similar to observations in lymphocytes, suggesting the Cd-mediated effects are not a cell-specific phenomenon [69,70]. Increased expression of various markers for apoptosis and oxidative stress (nuclear condensation, DNA fragmentation, Bax integration, cytochrome c release, etc.) were reported in fibroblasts, oligodendrocytes and lymphocytes [67,69,70]. Further strengthening the role of Cd-mediating mitochondrial changes leading to oxidative stress damage and apoptosis, Belyaeva et al. [71] report mitochondria obtained from rats showed mitochondrial swelling and transition pore changes following exposure to Cd. Changes in mitochondrial respiration can lead to the utilization of different pathways to generate ATP (aerobic to anaerobic respiration) [72]. Collectively, it is clear that Cd exposure alters mitochondrial function and increases Fas death receptor activation. The combination of these two actions results in a cascade leading to cellular damage (promoting tumor formation) or death. A schematic presentation of Cd-mediated cellular effects is depicted in in Figure 1.

Compared to other organ-based cell lines, there are limited numbers of studies focusing on the actions of Cd on thyroid cell lines in vitro. The use of other organ-derived cell lines to determine Cd-mediated toxicity has yielded increasing evidence to support the role of Cd-mediated toxicity. A consensus, among these studies, is that the mitochondria are a vital organelle in mediating Cd-related toxicities. In vitro studies with the ‘8505C’ thyroid cell line have demonstrated Cd-induced toxicity at relatively low Cd concentrations [73,74]. Observed toxicity was reversed when cell media was supplemented with either calcium or selenium, suggesting interference with Cd uptake. Cd-induced toxicity in thyroid cells also appears to be due to alterations in apoptosis, as has been observed in other cell lines [75,76]. Apoptosis induced by Cd was negatively correlated with metallothionein concentration [76]. Induction of heme oxygenase-1 and p21 are also vital for Cd-induced apoptosis [75]. Inhibiting p38 mitogen-activated protein kinase (p38MAPK) or extracellular-regulated kinase (ERK) significantly attenuated Cd-induced apoptosis [75]. Although not as numerous, the thyroid cell line-based investigation into Cd-induced toxicity has supported previous reports in other organ systems suggesting similarity in the pathways involved. Cd exposure is clearly toxic to thyroid cell lines with an LD50 range of 10–30 μM [76], with alterations in apoptotic pathways being a major source of cellular change potentially leading to tumor development. More studies are needed with varied thyroid cell lines to further delineate the pathways involved with Cd-mediated cellular changes and tumor development.

### 3.2. Secondary Endocrine Toxicity

Thyroid hormones are metabolized in peripheral tissues (by deiodination, conjugation, deamination, and decarboxylation), and alterations in their metabolism may significantly influence the thyroid function, especially the extra-thyroidal production of T3 from T4 [57,77,78,79]. Contrary to T4, most of the circulating T3 originates from the extra-thyroidal tissues. The rodent study investigating dose-response relationship between Cd and thyroid hormone levels proposed even more pronounced effects of Cd at the extrathyroidal level than on the gland itself [52]. Namely, the exposure to the lowest dose of Cd (0.3 mg Cd/kg b.w.) resulted in significantly reduced levels of T3, while T4 levels were significantly diminished only at higher doses (1.25 mg Cd/kg b.w.).

Some possible mechanisms of Cd secondary, extrathyroidal mechanisms, of toxicity are; altered hepatic metabolism of T4 or T3, changes in thyroid hormone peripheral receptors, or modulations in HPT axis functioning. Peripheral deiodination of T4 to T3 takes place mainly in the liver and is dependent on 5′-monodeiodinase (5′-D), a seleno-enzyme containing a selenocysteine residue as its active site [79]. Cd can inhibit 5′-D activity through binding to sulfhydryl groups of this enzyme. Also, this enzyme binds to cell membranes and Cd is shown to induce lipid peroxidation in the liver and blood [19,80,81]. Furthermore, Cd and Se interactions proved in many studies [82] could be one of the possible mechanisms of Cd-induced enzyme inhibition. In addition to changes in 5′-monodeiodinase activity, Cd exposure has been shown to induce the activity of UGT in the liver resulting in increased metabolism of T3 and T4 (lowering effective plasma concentrations) [43,48]. Extra-thyroidal disruption of thyroid function can also happen through inhibition of the thyroid hormone receptor (TRβ) gene expression, which was shown in Chinese toad tadpoles larvae exposed to different Cd concentrations [64]. Changes in circulating free T3 and T4 may be due to alterations in the function of Tg, the thyroid hormone binding globulin. Free, unbound, hormone is the active form of the hormone and there is evidence that suggests increases in Tg expression leads to reduced free levels of T3 and T4 [10]. The positive correlation between Cd and Tg levels was most pronounced in females. Changes in pituitary TSH secretion caused by Cd discussed earlier [46,47,48,49,50,51] could be the consequence of Cd effects on pituitary secretion and are also regarded as Cd secondary effects. Moreover, in a study in proestrous rats, it was shown that Cd accumulates in various brain regions responsible for the control of pituitary hormones release [83]. As previously mentioned, Cd also induces thyroid autoimmunity that can be conjectured to be associated with its ability to activate estrogen receptors [10], which could be one more Cd-mediated extra-thyroidal toxic mechanism. The study using a rat model of preeclampsia, autoimmunity-related disease supported this hypothesis [84]. A low dose of intraperitoneally injected CdCl_2_ on 9–14 gestational days increased immunoglobulin production and the expression of cytosine deaminase in mature B cells, which when upregulated is directly related to autoimmune diseases.

To the point, Cd is shown to produce direct effects on the thyroid gland, influence the central nervous system and poses peripheral extraglandular effects. However, all these mechanisms are related and often closely associated. Hence, sometimes it is difficult to determine which of the mechanism(s) is responsible for the observed effect. Furthermore, in-depth molecular studies on mechanisms of Cd influence on thyroid function are essential to ascertaining the evolution of Cd-related thyroid disorders.

## 4. Cadmium Nonmonotonic Effects Related to Thyroid Dysfunction

Over the past few years, there has been an increasing interest in non-monotonic responses to environmental pollutants and endocrine disrupting chemicals such as Cd in toxicological studies. These effects manifest in biphasic, bimodal or nonlinear dose-response relationship curves for various endpoints of thyroid dysfunction.

Cd-induced thyroid disruption was studied in Chinese toad (*Bufo gargarizans*) embryos by Wu et al. [85] at a range of exposure levels (5–500 μg Cd L^−1^). Results of the study revealed a bimodal inhibitory effect of Cd in growth and development of embryos, which the authors attributed to increased TH concentration. Although the frequency of embryo malformations was dose-dependent, Cd exposure led to the decrease of thyroid hormone receptors (THR) and the increase of T3 levels, differently below and above 100 μg Cd L^−1^. Researchers reported that thyroid hormone receptors (TRα) mRNA levels were significantly decreased in embryos exposed to all concentrations, while for embryos exposed to 100 μg Cd L^−1^, TRα mRNA levels were not altered. Changes in TRα mRNA levels in combination to the fact that 500 μg L^−1^ Cd down-regulated level of Dio3 expression (Dio3 inactivates T4 and T3) and up-regulated levels of Dio2 expression (Dio2 converts T4 to T3) lead to the increased TH concentrations. Similar results were published earlier by Sharma and Patiño [86] who while investigating Cd effects on Xenopus laevis tadpoles, reported a trend for a biphasic response of developmental rate to Cd exposure and suggested that Cd-evoked reduced the activity of the thyroid gland, which may be responsible for the developmental arrest. Moreover, progesterone, which has recently been found to regulate THR expression [87], exhibited a biphasic dose response to Cd concentrations [88]. Low doses of Cd increased progesterone levels, whereas high Cd doses reduced progesterone synthesis.

Detoxification pathways are similar for most systems, including the thyroid. Recent literature, tackling the topic of biphasic, or non-monotonic, responses to Cd exposure in vivo and in vitro, suggest that non-monotonic responses are linked to major detoxification mechanisms. Different endpoints investigated from zebrafish mortality [89] to antioxidant enzyme activity reveal non-linear or bimodal responses and imply hormetic phenomena. Cadmium induced a hormetic response to the activity of antioxidant enzymes, with increased activity at low Cd doses and enzyme inhibition at high Cd doses in *Eisenia fetida*. These responses were characterized by an inverted U shaped curve [90]. In vitro studies researching the endocrine disrupting effects of Cd revealed nonmonotonic effects on the expression of angiogenesis genes [91] and metallothionein expression in the intestine and kidney of female rats [92]. Furthermore, Jiang et al. [93] experimenting with human embryo lung fibroblast cells, reported a biphasic effect of Cd on cell proliferation. Specifically, that low Cd concentrations induces pathways that promote cell proliferation whereas high Cd concentrations inhibit cell proliferation.

## 5. Cadmium and Thyroid Cancer

The role of Cd as a human thyroid carcinogen is unclear, although some studies provide evidence of its possible role in the etiology of this disease. As recently reviewed by Vigneri et al. [13], epidemiological studies have pointed to higher thyroid cancer incidence in the volcanic areas with non-anthropogenic pollution with heavy metals, among which is Cd. Furthermore, determination of Cd concentrations in autopsy samples of retired Idrija residents and mercury mine workers revealed disproportionately high Cd concentrations in kidney cortexes and thyroid glands, pointing to possible bioaccumulation in the thyroid gland [94]. Chung et al. [95] examined 92 Korean women undergoing thyroidectomy to evaluate the association between blood and tissue levels of heavy metals to different stages of thyroid cancer. The study showed that the higher tissue Cd levels were associated with more advanced stage of thyroid cancer. They have identified the chronic Cd accumulation in thyroid tissue as one of the aggravating factors for thyroid cancer progression. Since their results demonstrated that only tissue Cd concentrations, not blood Cd concentrations, as positive collates to thyroid cancer stage, they have indicated the Cd carcinogenic effect to be stronger at the cellular level of the gland than that at the peripheral level of thyroid function.

In vitro examination of the role of Cd is limited. Few studies have investigated the actions of Cd on thyroid cell function. The majority of the existing data has utilized tissue samples from humans or rodents to assess Cd effects on thyroid function. Cellular damage may result from chronic Cd-mediated action at the mitochondria and generation of free radicals, leading to multiple thyroid pathologies [63,96]. The hormonal response to these cellular changes is a reduction in T3 and T4 levels and a blunted TSH response to falling levels of T3 and T4 [97]. There have been numerous studies that describe the action of Cd on tumor cells obtained from a variety of tissues such as breast [98,99,100,101], liver [102,103], pancreas [26,104], skin [105] and gastrointestinal tract [106] among others. Reports of direct Cd effects on thyroidal cells has been limited at best and the molecular-cellular mechanisms of Cd-mediated toxicity are unknown [107]. Interactions with the peripheral deiodinase enzyme have been reported with reductions in T3 production from T4 [108]. Using a two-hybrid bioassay to assay the actions of drinking water contaminants, including Cd, Li et al. [109] demonstrated that at low concentrations, Cd acted as a thyroid receptor antagonist. Cd was the most potent antagonist of the metals examined with a potency series of Cd > Hg > Zn [109]. In addition to the blockade of the thyroid receptor, in the yeast two-hybrid assay, Cd exposure reduced the proliferation of cells to a concentration of 1.0 μM. Once Cd moves to the intracellular compartment, multiple actions occur in thyroid tumors. ARO cells (Anaplastic Thyroid Carcinoma) are a thyroid tumor line that has been characterized for in vitro studies and when exposed to 20 μM Cd, demonstrate marked changes in metallothionein content and apoptotic activity [110]. Whether the reported Cd effects on ARO cells are direct or indirect, remain to be elucidated. Cd exposure resulted in a robust increase in intracellular calcium, which in turn will increase the phosphorylation of ERK1/2 [107,110] and increase cell growth from the G1- to S-phase [110]. The various isoforms of metallothionein are responsible for binding metals, either as a transport and storage mechanism (such as for copper, zinc, etc.) or for detoxifying the effects of other metals (such as mercury, Cd, arsenic, etc.). Induction of metallothionein 1 and metallothionein 2 expression following Cd exposure may be a response to detoxify the cell from raised Cd and protect against mitochondrial damage or increased ROS formation [107]. In addition to the increased expression of metallothionein, elevated intracellular calcium also results in the increased phosphorylation and activation of the PI3K/Akt signaling pathway [107], which promotes apoptosis. To counter pro-apoptotic pathways, p38, ERK and JNK are activated as survival pathways. Therefore, there is a delicate balance between apoptosis and survival, and the tipping of this balance will determine whether the cell survives or dies. Reports have indicated that thyroid cancer is higher in women, suggesting a potential hormonal influence on the development of thyroid cancer [111]. Thyroid cells express the estrogen receptors ERα, ERβ, and GPER [112,113,114]. Cd has been termed a ‘metalloestrogen’, although the terminology is unclear, Cd has been shown to elicit estrogen-like activity [22,98,101,115]. Zhu et al. [116] demonstrated that cells expressing GPER that are exposed to Cd showed an increase in cell proliferation and migration. Increased migration and proliferation are reversed when a GPER antagonist was added to the culture. These findings suggest Cd-mediated effects in thyroid tumors are due in part to activation of estrogen receptors, particularly the GPER and may explain in part gender differences in the incidence of thyroid cancer.

The epigenetic analysis in tumor pathology is becoming more mainstream, with methylation being the primary epigenetic mechanism in carcinogenesis, [117] resulting in unique methylation signatures between different types of tumor [118]. In thyroid cancer, several genes are downregulated via hypermethylation, including HOXD10 and MT1G [117,119]. HOXD10 has previously been identified as a potential tumor suppressor in several cancers, including breast, lung, and gastrointestinal cancers [117]. Evidence indicates HOXD10 suppresses the migration of thyroid tumor cells and promotes apoptosis, but is consistently and significantly hypermethylated in carcinogenic tissue samples [117]. Of particular interest, MT1G encodes metallothionein heavy metal binding proteins that are active in homeostasis by detoxifying heavy metals and scavenging free radicals [119]. Metallothionein is protective against metal toxicity by binding metals, such as Cd, and preventing free metals from damaging cellular structures [120,121,122]. Cd metal specifically, could be essential for the induction or promotion of thyroid cancer. Although it does not bind to DNA directly, Cd can operate through epigenetic mechanisms, including the interference of DNMTs, disruption of DNA repair mechanisms, and ROS production [26]. There is evidence of upregulation of the c-fos proto-oncogene with Cd exposure, which interferes with p53 tumor suppressor function [26]. This change, along with increased oxidative damage coupled with a decrease in apoptotic pathways associated with Cd exposure, could provide a potential mechanism for thyroid carcinogenesis [26].

As more retrospective studies attempt to draw correlations between Cd levels in blood and organs with tumor progression, a better understanding has developed regarding Cd as a thyroid carcinogen. Animal studies have supported the findings from human studies, further strengthening the correlation between Cd concentration and alterations in thyroid hormone synthesis and function. The cellular mechanisms proposed for Cd-mediated toxicity to thyroid cells is similar to mechanisms reported in other organ systems that lead to tumor formation. The three predominant toxic mechanisms are (1) alterations in the redox status of the cell and generation of free radicals, (2) changes in the apoptotic pathways necessary for normal cell survival and (3) epigenetic changes that would interfere with the proper and vital functioning of DNA and RNA. The role of Cd as a carcinogen in the thyroid is strong. More in vitro work must be performed to expand our understanding of Cd-toxicity on the cellular level in thyroid cancer.

## 6. Conclusions and Future Research Perspectives

The body of evidence demonstrates that Cd is, and will continue to be, one of the major health concerns for years to come. The ability of Cd to bioaccumulate and remain persistent in the environment for many years increases the health hazards associated with Cd exposure. Available epidemiological and experimental data indicate that Cd can disrupt thyroid gland function even at low environmental doses, both at target gland and extra gland level. The role of Cd in autoimmune disease and in thyroid cancer has been demonstrated. Given the high prevalence of general population exposure to this metal, future in-depth molecular studies on the Cd role in thyroid disruption and carcinogenesis are of high priority. Furthermore, studies examining parallel exposures to low concentrations of various putative toxicants are needed since mixture thyrotoxicity cannot be easily predicted. Hence, mixture toxicity, especially in term of endocrine disruption, should be research imperative. Such studies would provide important input for future prevention-oriented policy actions and intervention measures and are of great importance not only for researchers, regulators and healthcare providers, but for the public as well.

## Figures and Tables

**Table 1:** Summary of studies analyzing effects of Cd on thyroid function in animals. ♂, male; ♀, female.

Cd Concentrations or Dose(s)	Exposure Duration	Species	Effects	Method of Quantifying	Reference
0.4, 0.8 and 2.4 mg Cd/L	2, 4, 24 or 96 h	juvenile rainbow trout (*Oncorhynchus* *mykiss*)	T4 ↑ (2–4 h exposure)	radioimmunossay kits	[41]
0.4 and 0.8 mg Cd/L	1 week	juvenile rainbow trout (*Oncorhynchus* *mykiss*)	T4 ↓	radioimmunoassay kits	[41]
25 mg CdCl_2_/L	24, 48, or 96 h	tilapia (*Oreochromis niloticus*)	plasma T3 ↓	immunoassay	[42]
CdCl_2_	7, 17 and 28 days	catfish (*Clarias batrachus*)	thyrotropin inactivation plasma thyroid hormones ↓	/	[43]
0, 0.5 and 2.5 mg Cd/L	96 h	Chinese rare minnow (*Gobiocypris rarus*) larvae	whole-body of fish thyroid hormones ↓ (2.5 mg Cd/L) tireoglobuline ↑	enzyme-linked immunosorbent assay (ELISA)	[44]
10 and 25 μg Cd/L	30 days	adults rainbow trout (*Oncorhynchus* *mykiss*)	plasma T4 ↓; T3 ↓	commercial radioimmunoassay kits	[45]
1 and 5 g μg Cd/L	30 days	juvenile rainbow trout (*Oncorhynchus* *mykiss*)	no effects plasma T4; T3	commercial radioimmunoassay kits	[45]
200 ppm Cd (as CdCl_2_)	35 days, via drinking water	rats, *Wistar* albino ♂	relative thyroid weight ↑ serum TSH ↑ serum T4 ↓	commercial radioimmunoassay kits	[46]
50 mg Cd/L (as CdCl_2_)	4 weeks	rats, Sprague-Dawley ♂	serum T4 ↓, T3 ↓ serum TSH ↑	commercial kits	[47]
5, 10, 25, 50 or 100 ppm (as CdCl_2_)	1 month, via drinking water	rats, Sprague-Dawley ♂	plasma TSH ↑ (5, 25 and 100 ppm)	radioimmunoassay	[48]
25 and 50 mg/L (CdCl_2_)equivalence is 1.5 and 3 mg CdCl_2_/kg bw/day	30 days, via drinking water	rats, Sprague-Dawley ♂	TSH ↑ (at 12:00 and 16:00 h with the 25 mg/L and at 08:00 h with the mg/L)	specific double antibody radioimmunoassay	[49]
0.55 and 2.19 mg/L (as CdCl_2_)	12 weeks, via drinking water	rats, albino ♂	T4 ↓, T3 ↓, TSH ↓	radioimmunoassay method	[50]
5 and 50 mg Cd/L (as CdCl_2_)	12 months	rats, *Wistar* ♀	serum T4 ↓ (50 mg/L); no effects serum T3 and TSH	radioimmunologically	[51]
0.3, 0.6, 1.25, 2.5, 5 and 10 mg Cd/kg bw/day	28 days	rats, *Wistar* ♂	T3 ↓, FT3 ↓, T4 ↓, FT4 ↓ (BMDL_5_ 0.059 mg/kg bw/day for T3; BMDL_5_ 0.141 mg/kg bw/day for FT3; BMDL_5_ 0.365 mg/kg bw/day for T4; BMDL_5_ 0.354 mg/kg bw/day for FT4)	electrochemiluminescent immunoassay (ECLIA)	[52]
2.5, 7.5 and 15 mg Cd/kg bw/day	28 days	rats, *Wistar* ♂	T4 ↓, FT4 ↓, TSH below the limit of quantification	commercial tests, Elecsys analyser	[60]
